# Patient‐centred care is a way of doing things: How healthcare employees conceptualize patient‐centred care

**DOI:** 10.1111/hex.12615

**Published:** 2017-08-25

**Authors:** Gemmae M. Fix, Carol VanDeusen Lukas, Rendelle E. Bolton, Jennifer N. Hill, Nora Mueller, Sherri L. LaVela, Barbara G. Bokhour

**Affiliations:** ^1^ Center for Healthcare Organization and Implementation Research (CHOIR) VA Health Services Research and Development Service Bedford MA USA; ^2^ Boston University School of Public Health Boston MA USA; ^3^ Evaluating Patient‐Centered Care Bedford MA USA; ^4^ Center for Evaluation of Practices and Experiences of Patient‐Centered Care Hines IL USA; ^5^ Center for Healthcare Studies Institute for Public Health and Medicine General Internal Medicine and Geriatrics Feinberg School of Medicine Northwestern University Chicago IL USA

**Keywords:** healthcare workers, organizational change, patient‐centred care, qualitative research

## Abstract

**Background:**

Patient‐centred care is now ubiquitous in health services research, and healthcare systems are moving ahead with patient‐centred care implementation. Yet, little is known about how healthcare employees, charged with implementing patient‐centred care, conceptualize what they are implementing.

**Objective:**

To examine how hospital employees conceptualize patient‐centred care.

**Research Design:**

We conducted qualitative interviews about patient‐centred care during site four visits, from January to April 2013.

**Subjects:**

We interviewed 107 employees, including leadership, middle managers, front line providers and staff at four US Veteran Health Administration (VHA) medical centres leading VHA's patient‐centred care transformation.

**Measures:**

Data were analysed using grounded thematic analysis. Findings were then mapped to established patient‐centred care constructs identified in the literature: taking a biopsychosocial perspective; viewing the patient‐as‐person; sharing power and responsibility; establishing a therapeutic alliance; and viewing the doctor‐as‐person.

**Results:**

We identified three distinct conceptualizations: (i) those that were well aligned with established patient‐centred care constructs surrounding the clinical encounter; (ii) others that extended conceptualizations of patient‐centred care into the organizational culture, encompassing the entire patient‐experience; and (iii) still others that were poorly aligned with patient‐centred care constructs, reflecting more traditional patient care practices.

**Conclusions:**

Patient‐centred care ideals have permeated into healthcare systems. Additionally, patient‐centred care has been expanded to encompass a cultural shift in care delivery, beginning with patients' experiences entering a facility. However, some healthcare employees, namely leadership, see patient‐centred care so broadly, it encompasses on‐going hospital initiatives, while others consider patient‐centred care as inherent to specific positions. These latter conceptualizations risk undermining patient‐centred care implementation by limiting transformational initiatives to specific providers or simply repackaging existing programmes.

## 1 INTRODUCTION

Patient‐centred care (PCC) is growing in prominence. Balint first described PCC as “understanding the patient as a unique human being.”^1^ During the 20th century, PCC became a focus of healthcare systems with its promise to increase patient satisfaction and improve outcomes.^2^ A growing body of evidence has linked PCC practices with improvements in a variety of health conditions^3^, increased adherence^4^, decreased healthcare utilization^5^, better ratings of care^6^ and improved quality of care.^7^ By 2001, The Institute of Medicine identified PCC as a key aspect of high‐quality care.^8^ PCC is now ubiquitous internationally^9^ and extends beyond the provision of health care into medical education, law and quality improvement.^10^, ^11^ Moreover, PCC has increasingly become part of the research lexicon. A PubMed search (2016) for “PCC” produces over 20 000 results, with over 2000 publications annually.^12^, ^13^, ^14^

While the term PCC has become pervasive, consensus is lacking on what constitutes PCC.^15^, ^16^, ^17^, ^18^, ^19^ Most providers, policymakers and researchers agree that PCC represents a shift from a traditional, paternalistic, provider‐driven and disease‐focused approach towards one that fully integrates the patient's perceptions, needs and experiences, into every phase of medical consultation, treatment and follow‐up.^19^ In their oft‐cited literature review, Mead and Bower describe PCC as encompassing five conceptual dimensions: the *biopsychosocial perspective*;* patient‐as‐person*;* sharing power and responsibility*;* therapeutic alliance*; and *doctor‐as‐person*.^20^

Presently, there is limited research on hospital employee perceptions of PCC.^21^, ^22^, ^23^, ^24^, ^25^ To date, this research has been limited to studies with nurses or providers outside of the US^23^, ^24^ or focused on only a small subset of employee perceptions of patient‐centred care^22^, ^25^. As the research community continues to examine PCC^15^, ^17^, ^18^, healthcare systems are actively implementing initiatives.^26^, ^27^ Substantial efforts are underway, for example, to transform the US Veteran Health Administration (VHA) to a patient‐centred healthcare system. Several system‐wide policies are in place which emphasize the delivery of “personalized, proactive, and patient‐driven” care.^28^ To facilitate advancement of these PCC initiatives, VHA established the Office of PCC and Cultural Transformation (OPCC&CT), in 2010, to help facilities transform.^29^

While researchers and policymakers may be invested in PCC, it is up to hospital employees to enact it in practice. Their conceptualizations of what constitutes PCC shape how initiatives are developed, implemented and enacted. Therefore, we sought to understand how hospital employees conceptualize PCC.

## 2 METHODS

### 2.1 Setting

We conducted a qualitative study in the US's largest integrated healthcare system, as they embarked on implementing PCC. The VHA provides care to US military Veterans at over 150 medical centres across the US. As part of a larger study examining PCC implementation, we visited four VHA facilities designated as PCC “Centers of Innovation” (COIs). The COI designation was awarded to these four centres based on the facility's prior leadership and support for PCC development, innovation and implementation. The data were collected for quality improvement purposes to inform the field about best practices for PCC implementation. The Institutional Review Board (IRB) reviewed our work and designated our evaluation as IRB‐exempt. No formal consent was required; however, we provided verbal and written descriptions of the goal and scope of our evaluation work.

### 2.2 Participants

We worked closely with medical centre leadership, including the medical centre director or associate director and PCC coordinators, to identify individuals they considered critical to the implementation of PCC innovation. In addition to participating in interviews themselves, they identified a broad range of clinical and non‐clinical employees such as middle managers and front line staff for interviews. Potential participants were contacted via email explaining the purpose of the site visit and inviting participation. Interested participants were interviewed during the site visit.

### 2.3 Data collection

The interviews covered a range of topics. Two central questions that guided our analysis were as follows: “What do you think about when you hear the term PCC?” and “What are the key elements for care to be patient‐centered, from your perspective?” Our interview guide was designed to be used flexibly, depending on the participants' unique background and course of the conversation.

We audio‐recorded interviews with each participant's consent, between January and April 2013. Although most interviews were conducted individually, group interviews were conducted when the key informant brought others he/she felt were important to answering questions outlined in the email invitation. For example, at one site the integrative medicine lead invited other staff involved in the programme. Interviews lasted approximately one hour. We audio‐recorded interviews with participants' consent; when participants declined audio‐recording, we took detailed fieldnotes. Audio‐recordings were transcribed.^30^, ^31^ Transcripts and fieldnotes were entered into NVivo.^32^

### 2.4 Analysis

Data were initially coded using grounded thematic methods.^33^ Through group discussion, we examined how the PCC findings mapped to Mead and Bowers' 5 constructs: the biopsychosocial perspective (broadening beyond a disease state to include the psychological and social domains); patient‐as‐person (understanding an individual's particular illness experience, in the light of the his/her individual life context); sharing power and responsibility (egalitarian doctor—patient relationship); therapeutic alliance (a professional doctor—patient relationship, which entails empathy, congruence and unconditional positive regard); and doctor‐as‐person (views the doctor as an integral part of a two person relationship).^20^ These definitions provided an a priori framework to organize our findings and to determine how employee conceptualizations aligned with the existing PCC literature. We categorized coded segments according to Mead and Bower and developed grounded codes for data that did not fit. We developed the codebook iteratively until consensus. GF led categorization, which was reviewed with the full team to come to agreement.

## 3 RESULTS

We conducted 77 interviews with 107 employees across the four medical centres (Table 1). In 13 instances, descriptions of PCC were absent from the data. Because of the flexible nature of the qualitative interview, some interviewees provided broad descriptions of PCC, while others focused on a specific initiative and therefore did not provide a broad description of PCC. We organized employee conceptualizations of PCC into three broad categories in relation to Mead and Bowers' model. (i) Well aligned: the conceptualization mapped onto one of Mead and Bowers' five domains. (ii) Extended: the conceptualizations were highly congruent with Mead and Bower, extending it into organizational. (iii) Unaligned: the conceptualizations were vague or did not promote PCC transformation. Below, and in Table 2, exemplars are provided.

**Table 1:** Employee by role

Role	N
*Senior Medical Centre Leadership*	
Medical Centre Director Assistant Director Senior Management Team Chief of Staff Associate/Deputy Chief of Staff Chief Financial Officer Nursing Leadership	13 interviews; 22 people
*Middle Management*	
Patient‐Centred Care Coordinators and Leaders Patient‐Centred Care Committee Members Service Chiefs Voluntary Service Information Technology Pharmacy Engineering Environmental Management Services Specialty Clinical Service Anaesthesiology Home‐Based Primary Care Integrative and Alternative Medicine Managers Educational Leaders Health Promotion and Disease Prevention Coordinator	23 interviews; 41 people
*Front Line Providers*	
Psychologists Physicians Recreational Therapists Nurses Dieticians Social Workers	34 interviews; 37 people
*Other Staff*	
Systems Redesign Coordinators Architects Interior Designers Public Affairs Officers Information Technologist Program Specialist	7 interviews; 7 people
Total	N = 77 interviews or group interviews; 107 people

**Table 2:** Employee conceptualizations of patient‐centred care, organized by alignment with empirical literature

Theme	Exemplary quote
Well aligned^a^	
Biopsychosocial perspective	“[Medical providers] go into the healthcare field with a certain perspective, but patients have an entire family and environment that is affected by their health”*—Social Worker*
Patient‐as‐person	Being patient‐centred means “there's that personal touch, and personal communication, so they're not just name and last 4 [of their social security number]. When they come in, people know who they are, recognize ‘em…The clerk would know those patients, and would be able to say, you know, ‘Hi Mr. Smith,’ and remember something personal about that person. …You know, oh, you've got a new grandchild, how's it going, or, you know, or, or your son's graduating from high school, I remember you told me about that”*—Patient‐Centred Care, Leadership*
Sharing power and responsibility	“Patients call the shots. Doctors are helping them achieve their goals.”*—Lead Nurse*“What the doctor thinks is the best course of treatment is not what the patient necessarily selects”*—Chief of Information Technology*
Therapeutic alliance	Patient‐centred care requires “taking the time to know and engage the patient”*—Senior Management Team*
Doctor‐as‐person	“It's about empowering providers in the work setting, so that they have the best tools available to them to pursue wellness, both personally and professionally, so that they are able to partner with patients”*—Psychologist*
Extending to the organizational level	
Cultural Shift	“Patient‐Centered Care is not a movement; perhaps individual initiatives are, but Patient‐Centered Care is a way of doing things”*—Senior Management Team*
Atmosphere enhances Patient‐Centred Care	“It's about how patients feel when they walk into a facility and how they are greeted by staff. Do they feel safe? Do they have trust in their care?”*—Chief of Specialty Care*
Unaligned	
What I've always done	In response to a request to define Patient‐Centred Care, a psychologist replied “everything we do [in mental health] is patient‐centered. I don't know what you mean”*—Psychologist*
Multifaceted	“Patient‐Centered Care is a broad umbrella for encompassing everything”*—Senior Management Team*

a Alignment with Mead and Bower's review of patient‐centred care literature.

Several participants began their descriptions by stating that patient‐centred means the patient is at the centre. They used this metaphor to discuss patients as a focal point for understanding an aspect of PCC. For example, after noting that health care should be approached with “the patient at the center,” a Chief of Staff described the importance of learning about patient goals and context “with the patient, from their perspective” instead of what is being “done to the patient.” At the organizational level, a Member of Leadership noted “historically…we've really designed healthcare not around the patient, we designed it around ‘What do the physicians need? What do the nurses need?’”

### 3.1 Well aligned

In this first category, the participant's description of PCC mapped on to one of Mead and Bower's five conceptual domains below.

#### 3.1.1 Biopsychosocial perspective

For this domain, participants' described PCC as broadening beyond a patient's disease to include psychological and social domains, and as being “focused more on the patient as a whole person,” Nurse Manager. Participants' characterizations recognized patients as more than just a disease, broadening care practices to include patients' life contexts, social support, personality and spirituality “because we see the patient as more than just a patient, [but as] a human being with different facets of their personality,” Health Psychologist.

#### 3.1.2 Patient‐as‐person

Participants described seeing the patient as a person and needing to understand patients' illness experiences, within the patients' unique lifeworld. Several interviewees describe specific instances where employees took into account a patient's unique circumstances. A Nurse described how instead of focusing conversations with patients with diabetes on haemoglobin A1c levels, a provider could ask what patients want to do in their life, such as attend a relative's graduation. This would then provide an opportunity to discuss diet and exercise as a means to be around for these events. A Program Specialist gave an example of using technology to facilitate a family gathering for a hospitalized patient who was unable to attend his 50th wedding anniversary party. A nurse tended to this patient's unique circumstances by prompting the hospital to allow the patient to attend the ceremony virtually.

#### 3.1.3 Sharing power and responsibility

In this domain, participants describe an egalitarian relationship between patients and providers, in contrast to traditional, paternalistic relationships. In our data, participants repeatedly characterized PCC as a partnership between patients and their providers: “patients shouldn't be kept at a distance, but should be equal partners in care,” Patient‐Centered Care Committee Member. Sharing power and responsibility involved learning from patients: “We need to determine what patients' goals are, as well as those of caregivers. …all this occurs *with* the patient, from their perspective. It is not done *to* the patient,” Lead Nurse. This required “listening to the patient… having the patient be part of the team… [patients] wanna be heard.” A key aspect of this “means listening to what the patients want and not making assumptions about what they want,” Pharmacist.

This shared approach to care necessitated “an awareness that patients can say no,” Physician. This provider explained how providing PCC meant allowing the patient to choose the care plan, even if that meant going against recommendation. Similarly, participants mentioned the importance of not focusing on things that patients do not want. A Nursing Director noted that it, “takes a cultural shift to move from the old practice of requiring [patients] to attend smoking cessation to giving [them] the choice about whether to attend smoking cessation and to focus instead on mitigating risks for those who choose to continue smoking.” She added that this means providers need to accept patient's life choices, which is a change for both providers and patients.

#### 3.1.4 Therapeutic alliance

Descriptions of PCC mapping to this domain described focusing on the interpersonal relationship between the patient and provider, emphasizing empathy and respect. An Integrative Medicine Manager characterized PCC as, “not just a matter of [clinical] tasks, but rather, a matter of relationships.” She went on to describe how the patient‐provider relationship could serve as a foundation for individualized care.

#### 3.1.5 Doctor‐as‐person

This final domain of Mead and Bower's captures the interactional aspect of PCC by recognizing the clinical encounter as consisting of the doctor and patient, and how they influence each other. This category is not as well reflected in our data, which had few in‐depth descriptions of clinical encounters. Instead, much of our data focuses on providers and staff as a necessary component of PCC: “To do patient‐centered care, you have to start with employees,” Patient‐centered care Coordinator. An Assistant Director at another site similarly stated: “Patient‐centered should be expanded to include ‘Staff‐centered.’” A Patient‐Centered Care Coordinator expressed concern that staff would “walk with their feet” and leave the organization and went on to explain that the facility had a responsibility to take care of employees too. Employees were seen as important because it was believed they could not provide PCC unless they were also treated in comparable ways: “My philosophy is, if you take care of the employees, they care of the patients,” Patient‐Centered Care Coordinator.

### 3.2 Extending to the organization

Many of our participants broadened their descriptions of PCC beyond Mead and Bower's focus on the patient‐provider dyad, to the organizational level (see model). These descriptions were seen as supporting PCC, and included having a welcoming, conducive facility with a strong PCC culture.

#### 3.2.1 Atmosphere enhances patient‐centred care

Another component of this extension into the organization entailed having a welcoming and healing facility. A Patient‐Centered Care Coordinator explained that PCC entailed the entire experience of getting health care, “finding a parking place, getting into the building, finding where they're going…” She went on to explain how this should extend into the patient's appointment, where “all their concerns are addressed and that they're provided education and resources…that can help them.” A Service Chief linked a patient's experience upon entering the facility to good customer service: “when you ask for directions, the person you ask will walk you where you need to go. In low‐end places, the person will simply point and walk away…Training employees in customer service will make a big difference to how the facility is perceived.” Likewise, several other participants likened this positive experience to good customer service: “It's really just being nice and adding that extra touch,” Recreational Therapist.

Moreover, some interviewees noted that while physical change was part of their PCC transformation, it had to extend beyond remodelling. A Director of Nursing stated that PCC is “Not just warm and fluffy, the smell of cookies and cinnamon buns,” suggesting that PCC must go beyond the easy, feel good initiatives. She went on to describe how PCC could improve patient satisfaction and clinical outcomes, but to do this, PCC needs to be culturally ingrained.

#### 3.2.2 Cultural shift

Leadership, in particular, linked PCC to a cultural shift. A Director of Nursing said, “The crux of patient‐centered care is that it's a cultural shift for every single staff member.” She went on to explain that, “implementing and spreading patient‐centered care, [is a] huge process, and involves all of us living and breathing it, not just going to committee meetings.” A member of a Senior Management Team at another site noted, “It will be truly patient‐centered when it's not called patient‐centered care,” implying that PCC needs to be part of everyday practice instead of something distinct.

### 3.3 Deviations from patient‐centred care constructs

In this final category, conceptualizations of PCC poorly aligned with Mead and Bower. In these descriptions, participants conceptualized PCC as part of their existing care practices or as being so nebulous that it encompassed everything.

#### 3.3.1 What I've always done

In contrast to the cultural shift, several participants described PCC as being what they had always done and thus inherently part of their training and existing practices. A few participants described PCC as “what nursing has subscribed to for years” a Service Chief, with training as a nurse, equated the entire discipline of nursing with PCC. Likewise, a Director of Nursing described how other positions were not aligned with PCC, “If you asked a ‘med‐surg’ nurse to define patient‐centered care, they might stumble with the answers because they're not as used to patient‐centered care. Critical care nurses wouldn't have a clue”.

#### 3.3.2 Multifaceted

Some participants, particularly those in leadership, had difficulty articulating PCC as a single construct, “patient‐centered care is so multifaceted,” Director of Nursing. Similarly, a Member of Leadership at another facility said PCC was “not definable. It's a feeling.” Having multiple components made PCC difficult to reduce to a single description: “There is not one definition of patient‐centered care, but many.” This Member of Leadership added that he would “never try to define patient‐centered care because it will always evolve into something else.” Moreover, other participants extended the definition of PCC to into all aspects of healthcare service. In one interview, an engineer described infrastructure changes, including purchasing a new, efficient boiler, and then said that these activities were patient‐centred because the hospital can save money that can then be used for the patients.

## 4 DISCUSSION

Transforming care to being more patient‐centred is a complex task for healthcare organizations. We found that while the term “patient‐centered care” is expanding in use within the VHA, oftentimes the conceptualizations of employees are not fully congruent with the PCC constructs described in the literature. The VHA's explicit push for PCC by VHA's Office of Patient‐Centered Care and Cultural Transformation offers an ideal setting in which to examine employee conceptualizations of PCC.^29^ In the four medical centres in our study, many employees discussed PCC in terms that were consistent with Mead and Bower's core concepts (biopsychosocial perspective; patient‐as‐person; sharing power and responsibility; therapeutic alliance; and doctor‐as‐person). Employees also extended PCC beyond the patient‐provider dyad, yet, these were still consistent with the broader PCC literature.^8^, ^17^, ^27^, ^34^ This tells us that key PCC constructs are understood by those implementing PCC. The leadership, managers and front line staff we spoke with are trying to create a healthcare organization that subscribes to the ideals of PCC.

**Figure 1:**
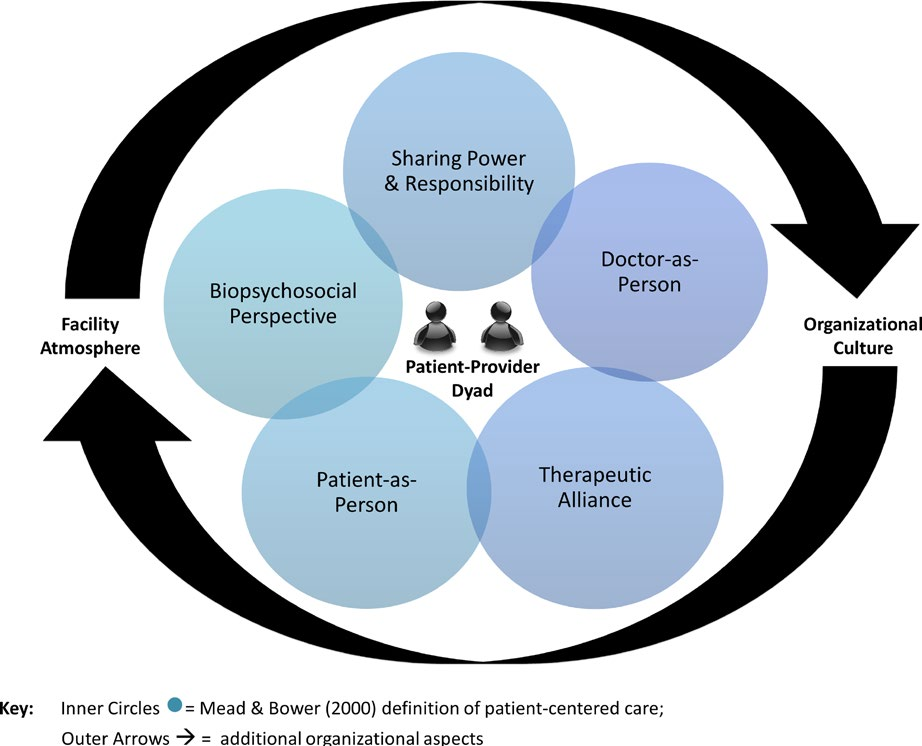
Healthcare Employee Model of Patient‐Centered Care

We found many employees' PCC conceptualizations extended beyond the traditional patient‐provider dyad, into the organization (See Figure 1). As Scholl's recent review of patient‐centredness found, policy‐level conceptualizations are lacking in the literature.^17^ However, the policymakers and front line providers we spoke with were clearly thinking about systems‐level implications of PCC. Our participants broadened the traditional definition by describing PCC as a cultural shift, imbued into care practices and organizational initiatives. Implementing discrete PCC programmes may not be effective at culture change, without a system‐level, multipronged approach^26^.

Participants discussed how physical surroundings can make patients feel comfortable, which while not reflective of current PCC literature, is highly aligned with organizational trends.^35^, ^36^ Yet PCC transformation does not end with physical changes. Our participants explained that PCC could not be achieved with an attractive facility alone—a therapeutic alliance or power sharing must be present. If PCC is conceptualized solely as the patient's experience when he/she enters a healthcare facility, an organization may lose focus on other key PCC components.^19^

Additionally, some participants talked about PCC in terms that were vague, broad or encompassed essentially everything. Notably, the characterizations of PCC that were unaligned with the PCC literature were primarily from leadership. Leaderships' broad conceptualizations may reflect their broad view of the organization and the need to have an overarching vision that encompasses all aspects of the facility. Yet this view may serve to water down implementation of core components of PCC. These amorphous conceptualizations index an intent to be patient‐centred, but without clearer definition do not provide adequate, actionable scaffolding upon which to implement cultural transformation. A broader understanding of PCC constructs can contribute to cultural transformation, but it may also limit the ability of leadership and policymakers to ensure PCC‐related goals are being achieved. Attempts to expand PCC constructs beyond the clinical encounter and into the organization are important for true transformation; however, when the conceptualizations seek to encompass everything currently subsumed under patient care, what happens in practice may not resemble what leadership and policy‐makers seek to achieve.

Some participants stated that PCC was what they had always done or was inherent to particular disciplines. These findings are consistent with others who found PCC politicized along disciplinary lines or perceived hierarchies in patient‐centredness.^37^, ^38^ Similarly, Gillespie et al^21^ found a tendency to re‐brand activities as patient‐centred, thereby negating the need to transform care. Where the problem lies is when some professionals misconceive PCC as something they have always done. In this instance, there are potential missed opportunities to understand how PCC may not in fact be what one has always done in their practice, or limited to specific roles. This viewpoint may limit these disciplines from re‐examining current practices or adopting new initiatives. When PCC is equated with a disciplinary approach, there is a risk of not making the cultural shift towards key aspects of PCC, such as sharing power, if there is the belief that the discipline is already patient‐centred. Engaging a range of employees is critical because hospitals are, by necessity, staffed with doctors, nurses, psychologists, pharmacists, managers, housekeeping and food service employees—all of whom interact with patients to some extent. Therefore, it is critical to have an organizational strategy, encouraging all employees to reflect on their own practice in relationship to the key components of PCC, as outlined by Mead and Bower, may facilitate greater insight and opportunity for growth^39^. PCC training could build on disciplinary principles aligned with PCC constructs, while providing clear examples of how PCC goes beyond current practice. Even brief trainings have been shown to be effective in enhancing PCC practice.^40^

We also found PCC being likened to good customer service. Linking PCC to customer service conflates the key differences between PCC and customer service oriented care.^34^ Epstein^18^, ^19^ cautions that PCC is more than just “giving patients what they want, when they want it.” And as Millenson notes, “what distinguishes patient‐centered care in its fullest sense from beneficence or better customer service is that it involves actions undertaken in collaboration with patients, not just on their behalf. It requires clinicians to appropriately share power even when that sharing feels uncomfortable.”^34^ Good customer service is important, but insufficient to truly providing PCC.

This study has limitations. Because the four VHA facilities identified key informants, we primarily spoke with employees directly involved in PCC, thus their conceptualizations of PCC might not be representative of employees less involved in PCC implementation. Additionally, the facilities in our study represent a subset of medical centres highly engaged in PCC; outside these centres, there is likely greater variation in how PCC is conceptualized. Further investigation is needed with other employees more removed from organizational change initiatives, yet still have frequent patient‐interactions, such as housekeeping or clerks. Finally, our data only represents how these employees perceive PCC. Observations of interactions between patients and healthcare employees are needed to determine what PCC looks like in practice. Future work should also address how patients understand patient‐centredness,^41^, ^42^ critical for shifting provider behaviours towards what patients want.

Nonetheless, our qualitative approach allows for an in‐depth examination of employee conceptualization of PCC in a large, integrated healthcare system. Our findings have important implications for other organizations trying to implement PCC transformation; broadening the definition of PCC beyond the clinical encounter to incorporate organizational elements is critical to culture change initiatives and implementation processes. We provide a caution, however, that when PCC is too broad, efforts may be watered down and true transformation hindered. Employees working in healthcare systems dedicated to PCC must have an appreciation of the model in order to align their efforts. Moreover, many organizations are trying to measure the impact of their PCC initiatives;^5^, ^26^, ^43^, ^44^ yet without clear definitions of PCC, they may not be measuring what they intend. Therefore, continuing to refine and expand upon models of PCC, such as Mead and Bowers', is essential to informing measurement.

In this paper, we focused on examining the perceptions of what constitutes PCC at the facility level because these conceptualizations shape how PCC initiatives are developed, implemented and enacted in a healthcare organization. Differing conceptualizations and scattered efforts will not serve to systematically advance PCC. In order to embed patient‐centredness in an organization's culture one needs to make it a systems property, which requires a clear understanding of the full range of factors that promote or impede PCC.^45^ As organizations move ahead with PCC implementation, they will need to consider if they are going to simply task specific disciplines with PCC practices or instead offer a more comprehensive, transformational organizational plan to infuse PCC throughout the facility. As Gillespie et al^21^ also note, there is a tendency to take the easy path and simply reframe existing work structures. Organizations that have succeeded in advancing PCC have adopted a comprehensive, organization‐wide approach linked to organizational strategy.^46^, ^47^ Communicating a strategic vision clearly and consistently to every member of the organization, with leadership engagement and support, is essential to developing an organizational approach to PCC.^46^, ^47^ Organizational change strategies that move beyond a shared vision, and engage staff and middle managers in developing work processes and interactions that implement that vision, are critical. It is the shared uptake of this vision that is most likely to result in synchronized efforts to transform the organizational culture to one where patients truly drive care.

## CONFLICT OF INTEREST

None disclosed.
